# 局部晚期非小细胞肺癌患者血清中Ape1/Ref-1、ICAM-1及IL-17A水平与放射性肺炎发生的相关性研究

**DOI:** 10.3779/j.issn.1009-3419.2018.05.05

**Published:** 2018-05-20

**Authors:** 雷鸣 郭, 高峰 丁, 文才 徐, 红 葛, 月 蒋, 寓非 陆

**Affiliations:** 450001 郑州，郑州大学附属肿瘤医院放疗科 Department of Radiotherapy, Affiliated Cancer Hospital, Zhengzhou University, Zhengzhou 450001, China

**Keywords:** 放射性肺炎, Ape1/Ref-1, ICAM-1, IL-17A, Radiation pneumonitis, Ape1/Ref-1, ICAM-1, IL-17A

## Abstract

**背景与目的:**

放射性肺炎主要表现为肺泡上皮及内皮细胞损伤、细胞炎性因子异常表达、纤维母细胞异常增殖及纤维基质合成，其发生与多种细胞因子水平异常相关，这些细胞因子亦可作为预测放射性肺炎发生的生物学指标。本研究评价局部晚期非小细胞肺癌（non-small cell lung cancer, NSCLC）同步放化疗患者放疗前和放疗后血清中脱嘌呤脱嘧啶核酸内切酶1（apurinic/apyrimidinic endonuclease 1/redox factor-1, Ape1/Ref-1）、细胞间粘附分子-1（intercellular adhesion molecules 1, ICAM-1）和白细胞介素-17A（interleukin-17A, IL-17A）水平的变化与放射性肺炎发生的相关性。

**方法:**

68例晚期NSCLC患者均行同步放化疗，每位患者正常组织受照剂量相同。在治疗前及治疗后第14周，采用ELISA法检测血清中Ape1/Ref-1、ICAM-1及IL-17A水平。应用美国肿瘤放射治疗协作组和欧洲肿瘤治疗研究协作组（Radiation Therapy Oncology Group/European Organization For Research and Treatment, RTOG/EORTC）诊断分级标准作为急性和晚期肺放射损伤分级标准评价，评价终点≥2级判定为放射性肺炎。

**结果:**

18例发生放射性肺炎，50例无放射性肺炎发生。放射性肺炎组放疗前后Ape1/Ref-1水平无显著性变化（*P* > 0.05）；放射性肺炎组与非放射性肺炎组Ape1/Ref-1水平无显著性变化（*P* > 0.05）。与放疗前相比，放疗后放射性肺炎组ICAM-1水平上调幅度比非放射性肺炎组ICAM-1水平上调幅度大（*P* < 0.05）。放射性肺炎组患者的IL-17A水平在放疗后显著上升（*P* < 0.05），而非放射性肺炎组IL-17A水平放疗前后水平无显著性变化（*P* > 0.05）；放疗后，放射性肺炎组IL-17A水平比非放射性肺炎显著性升高（*P* < 0.05）。相关性分析发现放疗前后ICAM-1的变化与放射性肺炎的发生无明显相关性，而IL-17A的变化与放射性肺炎的发生具有明显相关性。

**结论:**

晚期NSCLC患者同步放化疗时，在严格限制正常组织受照剂量的同时，血清IL-17A可能是放射性肺炎的预测因子。

## Abstract (en)

**Background and objective:**

The main manifestations of radiation pneumonitis are injury of alveolar epithelial and endothelial cells, abnormal expression of cytokines, abnormal proliferation of fibroblasts and synthesis of fibrous matrix. The occurrence of radiation pneumonitis is associated with multiplecytokine level abnormality. These cytokines can also be used as bio-markers to predict the occurrence of radiation pneumonitis. This study was to evaluate the correlation between the change of apurinic/apyrimidinic endonuclease 1/redox factor-1 (Ape1/Ref-1), intercellular adhesion molecules 1 (ICAM-1) and interleukin-17A (IL-17A) before and after radiotherapy and radiation pneumonitis for local advanced non-small cell lung cancer (NSCLC) patients with concurrent chemoradiotherapy.

**Methods:**

NSCLC patients (68 cases) were treated with concurrent radiotherapy and chemotherapy, every patient's normal tissue were controlled with a same radation dose. 68 local advanced NSCLC patients with concurrent chemoradiotherapy were detected the levels of Ape1/Ref-1, ICAM-1 and IL-17A in serum by ELISA before radiotherapy and in the 14th week after radiotherapy. Acute and advanced radiation pulmonary injury was graded according to Radiation Therapy Oncology Group/European Organization For Research and Treatment (RTOG/EORTC) diagnostic and grading criteria. Grade 2 or more radiation pneumonitis was taken as the main end point.

**Results:**

Eighteen cases out of 68 developed radiation pneumonitis, 50 of 68 cases have no radiation pneumonia development. There was no significant change of Ape1/Ref-1 levels before and after radiotherapy in radiation pneumonitis group (*P* > 0.05). There was no significant change of Ape1/Ref-1 concentration in serum after radiotherapy between radiation pneumonitis group and non-radiation pneumonitis group (*P* > 0.05). Compared with before radiotherapy, upregulation degree of ICAM-1 levels in radiation pneumonitis group was significantly higher than that in non- radiation pneumonitis group (*P* < 0.05). There was no significant change of IL-17A concentration before and after radiotherapy in radiation pneumonitis group, but after radiotherapy IL-17A concentration in serum were remarkably higher than that in non-radiation pneumonitis group (*P* < 0.05). Correlation analysis found that the change of ICAM-1 before and after radiotherapy has no obvious correlation with the incidence of radiation pneumonitis, and IL-17A change has obvious correlation with the incidence of radiation pneumonitis.

**Conclusion:**

On the basis of strictly controlling radiation dose on normal tissue, IL-17A in serum could be the predictive factors of radiation pneumonitis for local advanced NSCLC patients with concurrent chemoradiotherapy.

晚期非小细胞肺癌（non-small cell lung cancer, NSCLC）的预后差，目前同步放化疗是其标准的治疗方案。放射性肺炎（radiation pneumonitis, RP）是肺癌放射治疗的主要限制性并发症，也是重要的剂量限制性毒性反应，而同步放化疗亦会加重肺损伤^[1]^。目前，有关预测RP生物学标记物的研究较少，不利于个体化治疗方案的推进。因此深入探索RP发生发展过程中的相关生物学指标，对制定个体化的治疗方案具有非常重要的临床指导意义。

研究表明，放射性肺炎的主要特征有肺泡上皮及内皮细胞损伤、炎性因子异常表达、纤维母细胞增殖及纤维基质合成，与这些特征相关的细胞因子与放射性肺炎的发生紧密相关^[2-3]^。脱嘌呤脱嘧啶核酸内切酶1又称氧化还原因子-1（apurinic/apyrimidinic endonuclease 1/redox factor-1, Ape1/Ref-1）是一种在多种肿瘤中表达的多功能蛋白，其与放射治疗密切相关。研究发现，在放射性肺损伤进展过程中，放射导致的氧化应激使Ape1/Ref-1表达增加，但是肺损伤修复会消耗掉过量的Ape1/Ref-1蛋白。细胞间粘附分子-1（intercellular adhesion molecules 1, ICAM-1）是一种与细胞侵袭性相关细胞因子，在肿瘤放射过程中参与细胞损伤修复。白细胞介素（interleukin, IL）-17A是一种促炎症细胞因子，可促进关键放射性肺炎标记分子IL-6的表达。以上说明，Ape1/Ref-1、ICAM-1及IL-17A可作为肺炎潜在的生物学标记物。因此，我们检测晚期NSCLC同步放化疗患者放疗前和放疗后血清脱嘌呤脱嘧啶核酸内Ape1/Ref-1、ICAM-1和IL-17A水平变化，并研究其变化与放射性肺炎发生的相关性，探索放射性肺炎的预测因子。

## 1 材料与方法

### 1.1 一般资料

2014年12月-2015年12月接受同步放化疗的晚期NSCLC患者共68例，男36例，女32例，年龄52岁-76岁，中位年龄64岁。所有患者均经组织病理学或细胞学确诊，其中鳞癌32例，腺癌34例，大细胞癌2例；临床分期Ⅲa期32例，Ⅲb期36例。本研究经医院伦理委员会批准。

### 1.2 治疗方案

采用逆向调强放疗（intensity modulated radiation therapy, IMRT），热缩体膜或真空体架固定，CT模拟定位扫描，扫描层间距3 mm；在Varian Eclipse DX计划系统勾画靶区设计计划；根据ICRU50、62号报告对靶区和肺、食管、心脏、脊髓等重要器官进行勾画。大体靶区（gross tumor volume, GTV）为影像学上可见肿瘤（包括肺内病灶和纵隔淋巴结）；临床靶区（clinicaltarget volume, CTV）为GTV外扩6 mm（鳞癌）或8 mm（腺癌），支气管方向外放13 mm，包括部分淋巴引流区；计划靶区（planning target volume, PTV）为相应的CTV外扩6 mm-10 mm（综合考虑呼吸运动幅度和摆位误差等因素确定）；利用射野方向观视功能设计照射野；应用放射治疗计划剂量体积直方图（dose volume histogram, DVH）和等剂量曲线，结合患者肺功能状态和危及器官受量综合评价确定治疗计划；放疗处方剂量为60 Gy-70 Gy/30-35次/6周-7周；收集GTV的平均剂量、最大剂量、最小剂量，肺V5、V10、V20、平均剂量，正常组织并发症概率（normal tissue complieation probability, NTCP），食管V45，心脏平均受照剂量和脊髓最大受照剂量，共11个物理参数资料，确保每位患者放射的同时对正常组织无明显影响；同步化疗2个周期，采用化疗方案为顺铂+依托泊苷方案：顺铂50 mg/m^2^第1、8、29、36天，依托泊苷50 mg/m^2^第1天-5天、29天-33天。治疗结束后，无特殊情况每3个月随访1次。

### 1.3 分组

应用美国肿瘤放射治疗协作组和欧洲肿瘤治疗研究协作组（Radiation Therapy Oncology Group/European Organization For Research and Treatment, RTOG/EORTC）诊断分级标准作为急性和晚期肺放射损伤分级标准评价，有肺炎症状与体征，特别是胸部CT显示与射野区域一致的弥漫性片状密度升高影，诊断为放射性肺炎。0级：症状体征同治疗前比无明显变化；1级：轻微咳嗽、用力时气短；2级：持续性咳嗽需用麻醉性镇咳药、轻微用力时呼吸困难但静息时消失；3级：严重咳嗽、麻醉性镇咳药无效、静息时气短、临床或放射影像证实的急性肺炎、可能需要间断吸氧或应用皮质激素；4级：严重呼吸功能不全、持续吸氧或辅助通气。诊断放射性肺炎时必须排除肺内感染及肺内病变进展且相关症状分级至少较治疗前增加1级。观察终点：≥2级判定为放射性肺炎。根据是否发生放射性肺炎，将患者分为放射性肺炎组和非放射性肺炎组。

### 1.4 Ape1/Ref-1、ICAM-1及IL-17A的检测治疗前及治疗后

第14周清晨空腹静脉采血2 mL-3 mL，肝素抗凝4 ℃ 1200 r/min离心10 min后取血清存放于-80 ℃冰箱保存，检测时室温下融化后2 h内测量。Ape1/Ref-1、ICAM-1 ELISA试剂盒购自美国R & D公司，IL-17A ELISA试剂盒购自美国Chemicon公司，操作步骤严格按照说明书进行。

### 1.5 统计学方法

采用SPSS 19.0统计软件进行检验。计量资料采用均数±标准差（Mean±SD）表示，采用*t*检验。计数资料采用率（%）表示，采用卡方检验。以*P* < 0.05为差异有统计学意义。

## 2 结果

### 2.1 一般资料、随访及放射性肺炎发生情况

全组患者一般资料（性别，年龄，吸烟史等）与放射性肺炎的发病率无明显相关性（表 1）。随访至2016年12月，随访12个月-24个月，中位随访18个月，无失访。68例患者中有18例在放疗结束后15周内发展为放射性肺炎（2例发生在放疗结束后1周-2周，16例发生在放疗结束后6周-13周），其中2级10例，3级8例。

**1:** 患者一般资料 General information of patients

Variable	*n*	RP	NRP	X^2^ value	*P*
Gender				1.231	0.364
Male	36	9	27		
Female	32	9	23		
Age (yr)				0.576	0.453
≥60	37	11	26		
< 60	31	7	24		
Smoking				0.467	0.578
Yes	48	8	40		
No	20	10	10		
TNM stage				0.963	0.966
Ⅲa	32	10	22		
Ⅲb	32	8	2		
RP: indicates radiation pneumonitis group; NRP: indicates non-radiation pneumonitis group.					

### 2.2 放疗前后患者血清中Ape1/Ref-1、ICAM-1、IL-17A的水平变化

放射性肺炎组放疗前后Ape1/Ref-1水平无显著性变化（*P* > 0.05）；放射性肺炎组与非放射性肺炎组Ape1/Ref-1水平无显著性变化（*P* > 0.05）（表 2）。与放疗前相比，放疗后放射性肺炎组ICAM-1水平上调幅度比非放射性肺炎组ICAM-1水平上调幅度大（*P* < 0.05）（表 3）。放射性肺炎组患者的IL-17A水平在放疗后显著上升（*P* < 0.05），而非放射性肺炎组IL-17A水平放疗前后水平无显著性变化（*P* > 0.05）；放疗后，放射性肺炎组IL-17A水平比非放射性肺炎显著性升高（*P* < 0.05）（表 4）。

**2:** 两组放疗前后血清Ape1/Ref-1水平测定结果比较（ng/mL） Comparisons of the serum Ape1/ ref-1 level during different groups (ng/mL)

Variable	*n*	Pre-radiotherapy	Post-radiotherapy
RP	18	4.2±0.78	4.4±1.1
NRP	50	4.3±0.85	4.6±0.92
*t*		0.435	0.675
*P*		0.754	0.659

**3:** 两组放疗前后血清ICAM-1水平测定结果比较（ng/mL） Comparisons of the serum ICAM-1 level during different groups (ng/mL)

Variable	*n*	Pre-radiotherapy	Post-radiotherapy
RP	18	320.32±66.56	940.76±110.09
NRP	50	190.45±98.12	330.74±107.29
*t*		6.783	6.889
*P*		0.003	0.000

**4:** 两组放疗前后血清IL-17A水平测定结果比较（ng/mL） Comparisons of the serum IL-17A level during different groups (ng/mL)

Variable	*n*	Pre-radiotherapy	Post-radiotherapy
RP	18	5.6±0.90	12.4±0.98
NRP	50	5.5±1.09	3.12±0.97
*t*		5.884	9.835
*P*		0.063	0.000

### 2.3 IL-17A、ICAM-1变化和放射性肺炎发生的相关性

采用卡方检验进行相关性分析发现放疗后与放疗前相比血清中细胞因子IL-17A的变化和放射性肺炎发生具有明显的相关性；而ICAM-1的变化与放射性肺炎的发生无明显相关性（表 5）。

**5:** 血清中细胞因子水平变化与放射性肺炎发生的关系 The relationship analysis between cytokines levels and radiation pneumonitis

Variable	*n*	Pre-radiotherapy	Post-radiotherapy
RP	18	5.6±0.90	12.4±0.98
NRP	50	5.5±1.09	3.12±0.97
*t*		5.884	9.835
*P*		0.063	0.000
The increase of ICAM-1 greater than 50% indicates a significant difference.			

### 2.4 不同部位放射剂量相关参数情况

放射性肺炎组和非放射性肺炎组大体靶区（GTV）平均剂量、最大剂量、最小剂量，肺脏V5、V10、V20、平均剂量，正常组织并发症概率（NTCP），食管V45，心脏平均受照剂量，脊髓最大受照剂量等相关参数两组间差异均无统计学意义（表 6）。

**6:** 两组放射剂量相关参数比较 Comparison of relevant parameters between RP and NRP groups

Variable	RP (*n*=18)	NRP (*n*=50)	*t*	*P*
Lung				
V5 (%)	39.7±0.876	36.9±0.635	0.402	0.638
V10 (%)	17.3±0.217	15.3±0.403	0.689	0.198
V20 (%)	21.8±0.387	22.8±0.876	0.568	0.219
Average dose (Gy)	13.6±0.427	14.1±0.509	0.498	0.674
NTCP (%)	4.8±0.431	5.1±0.509	0.886	0.678
Esophagus				
V_45_ (%)	18.5±0.286	16.8±0.487	0.307	0.289
Heart				
Average dose	15.6±0.594	14.8±0.339	0.587	0.372
Spinal cord				
Maximum dose	33.2±0.209	37.2±0.501	0.129	0.309
GTV				
Minimum dose	45.9±0.786	48.1±0.387	0.413	0.682
Maximum dose	68.6±0.601	67.2±0.428	0.673	0.214
Average dose	56.3±0.682	57.2±0.361	0.683	0.483

## 3 讨论

晚期NSCLC是指不能手术的Ⅲa期和Ⅲb期病变，整体预后较差。目前，美国国家综合癌症网络（National Comprehensive Cancer Network, NCCN）等多个肿瘤研究组织均推荐同步放化疗作为晚期NSCLC的标准治疗模式。放射性肺损伤是肺癌放射治疗过程中的主要剂量限制性并发症，其在接受根治性放疗的肺癌患者中的发生率约为13%-37%^[1]^。放射性肺损伤的发生发展包括肺泡上皮及内皮细胞等的损伤、炎性因子的表达、纤维母细胞的增殖及纤维基质的合成等过程，这些过程是通过一系列的细胞及细胞间信号的表达、传导、放大、反馈及调节过程实现的^[2, 3]^。通过发现放射性肺损伤发生发展过程中的标记性细胞因子，有望为预测及早期发现放射性肺损伤的发生提供理论支持。诱导放射性肺损伤发生的因素可分为以下几个方面：放射物理学方面如肺受照剂量-体积因素等剂量学方面；患者基本状况如年龄、性别、既往肺病史、肺功能情况、吸烟史、营养状况、合并症；放射生物因素如患者内在易感性生物学的特征、某些细胞因子活性的不同及其在治疗中的变化与放射性肺炎的相关性不同。化疗药物可通过直接细胞毒损伤、氧化损伤、炎性反应介导损伤、胶原纤维合成与分解平衡破坏等机制导致肺损伤，同时放化疗药物还能阻碍机体对正常组织的修复。因此，同步放化疗在提高肺癌疗效的同时也增加了放射性肺炎的发生几率^[4]^。因而有必要探寻可靠的生物学标志分子来预测个体发生放射性肺炎的危险性，从而为个体化治疗方案的制定提供帮助。

Ape1/Ref-1是一种高度保守的多功能大分子蛋白，Ape1和Ref-1这2个具有截然不同功能的亚基逐渐整合到一起，形成了一个密不可分的功能复合体^[5]^。研究表明，Ape1/Ref-1在多种恶性肿瘤中过度表达，其与肿瘤的发生、发展及预后密切相关^[6-8]^。另外一些研究发现，电离辐射可使肺组织细胞处于氧化应激状态，从而诱导细胞代偿性合成Ape1/Ref-1蛋白，进一步调控参与细胞氧化应激反应的转录因子，而随着放射性肺损伤的进一步展，Ape1/Ref-1蛋白在肺损伤修复过程中的消耗超过了其在肺组织细胞的合成，从而造成放射治疗组Ape1/Ref-1水平明显低于未放射治疗组^[9]^。余舒亮等^[10]^人的研究报道指出，电离辐射可以刺激APE1/Ref-1蛋白过表达，并可使其发生由核内转向胞浆和出现表达先增高后降低的特征，表明Ape1/Ref-1可以作为放射性肺损伤防治的分子靶点，在放射性肺损伤发生、发展过程中可能起着损伤修复的重要作用。本研究中检测了68例晚期非小细胞肺癌患者在放疗前后以及放疗后，放射性肺炎发生组和未发生组组患者血清中Ape1/Ref-1的变化情况，均发现无论放疗前后放射性肺炎组和非放射性肺炎组相比，Ape1/Ref-1均无明显变化。因此，尽管Ape1/Ref-1与肿瘤的发生和放射性损伤密切相关，但其是否在预测放射性肺炎发生中有较为明确的指标作用，仍有待于进一步研究。

细胞间粘附分子-1（ICAM-1）是肿瘤细胞侵袭转移过程中非常重要的细胞因子，在多种类型的肿瘤组织中存在异常表达^[11]^。在关于不同组织器官的放射性损伤研究中，均发现ICAM-1与放射性损伤存在相关性^[12-15]^。但既往研究对患者基线水平一致性要求的不足，可能导致观察结果出现偏倚。因此，我们在本次研究中仅纳入基线水平无显著差异的患者，在排除了放射物理因素可能导致的偏倚以后，分别检测了放疗前后患者血清中ICAM-1水平，发现放疗前放射性肺炎组患者ICAM-1水平明显高于非放射性肺炎组，而在放疗后放射性肺炎组仍高于非放射性肺炎组，说明ICAM-1确实在肺癌患者血清中高表达，与之前研究一致，但通过相关性检验发现血清ICAM-1水平是否有显著性（≥50%）变化与放射性肺炎的发生并无相关性。依据临床标准，本研究规定50%为显著和不显著的标准，由于病例数较少，未能充分分析ICAM-1升高程度的不同与放射性肺炎的相关性，存在一定的局限性。

白细胞介素17A（interleukin 17A, IL-17A）作为一种促炎症细胞因子，参与了慢性炎症过程和自身免疫性疾病^[16-17]^。IL-17A能够增加人成纤维母细胞黏附分子-1（ICAM-1）的表达，刺激上皮细胞、内皮细胞或成纤维细胞产生IL-6、IL-8、G-CSF。研究发现细胞因子白介素-6（IL-6）可以作为预测放射性肺炎的重要因子^[3, 18]^。作为细胞产生IL-6的IL-17A，也可能成为预测放射性肺炎的重要因子。因此，本研究中也检测了患者放疗前后血清中IL-17A水平变化，发现放疗前放射性肺炎组与非放射性肺炎组血清中IL-17A水平无明显差异，放疗后出现明显的差异，相关性检验发现血清中IL-17A升高与放射性肺炎的发生成正相关。因此，IL-17A作为重要的促炎症细胞因子之一，可能对放射性肺炎的发生有较好的预测价值。

综上所述，本研究选取符合条件的68例晚期NSCLC同步放化疗患者，检测治疗前后患者血清中Ape1/Ref-1、ICAM-1及IL-17A水平，发现血清中IL-17A可能会为预测放射性肺炎的发生提供有力参考。但是放射性肺损伤的发生是多种细胞因子参与的复杂过程，单个细胞因子的变化可能无法精准反应这一过程。应用细胞因子预测放射性肺炎的发生时，需要对更多的细胞因子进行筛选，从而为寻找到更为有效的生物学参考指标提供理论依据。
